# Early Stage Lung Cancer Detection in Systemic Sclerosis Does Not Portend Survival Benefit: A Cross Sectional Study

**DOI:** 10.1371/journal.pone.0117829

**Published:** 2015-02-17

**Authors:** Jeremy B. Katzen, Kirtee Raparia, Rishi Agrawal, Jyoti D. Patel, Alfred Rademaker, John Varga, Jane E. Dematte

**Affiliations:** 1 Department of Medicine, Division of Pulmonary and Critical Care Medicine, Northwestern University Feinberg School of Medicine, Chicago, Illinois, United States of America; 2 Department of Pathology, Northwestern University Feinberg School of Medicine, Chicago, Illinois, United States of America; 3 Department of Radiology, Northwestern University Feinberg School of Medicine, Chicago, Illinois, United States of America; 4 Department of Medicine, Division of Hematology and Oncology, Northwestern University Feinberg School of Medicine, Chicago, Illinois, United States of America; 5 Department of Preventive Medicine, Division of Biostatistics, Northwestern University, Chicago, Illinois, United States of America; 6 Department of Medicine, Division of Rheumatology and Northwestern Scleroderma Program, Northwestern University Feinberg School of Medicine, Chicago, Illinois, United States of America; University of Texas Health Science Center at Houston, UNITED STATES

## Abstract

**Background:**

Systemic Sclerosis (SSc) is a rare connective tissue disorder associated with an increased risk of malignancy including lung cancer.

**Methods:**

A single center review of all cases of lung cancer in patients with SSc was conducted. Clinical, radiographic, and detailed pathologic data was collected. Risk factors were compared with our center’s SSc Registry. Cancer characteristics were compared with the National Cancer Institute SEER Cancer Statistics (NCI SEER) data.

**Results:**

17 cases were identified; the majority were females (82%) with the lung cancers diagnosed after the onset of SSc (88%). Tobacco use was identified in 65% of cases. Serologic testing showed 50% of cases were Scl-70 positive. Twelve cases had radiographic evidence of SSc lung involvement, however only 6 had restrictive physiology on pulmonary function testing. Thirteen cases had pulmonary nodules preceding lung cancer. Thirteen of the cancers were adenocarcinoma. Ten underwent molecular mutational profiling: 2/8 had KRAS mutation and 1/10 had EGFR mutation. More of the non-small cell lung cancers were diagnosed at localized disease (56%) than in the NCI SEER database. However, 5 years survival among the stage I cases was 25% versus an expected survival of 54%.

**Conclusions:**

The high proportion of adenocarcinomas seen in our study is different from that reported in the literature. Lung cancers were diagnosed at an early stage, likely due to our center’s practice of radiographic screening for SSc associated lung involvement, however this did not confer a survival advantage. A high proportion of patients who developed lung cancer had interstitial lung disease.

## Introduction

Systemic sclerosis (SSc) is a connective tissue disorder characterized by immune dysregulation and accumulation of extracellular matrix constituents resulting in organ dysfunction.[1] Pulmonary involvement is the leading cause of SSc-related mortality. However, cancers as causes of morbidity and mortality are of increasing interest.[2]

A number of population-based SSc cohort studies have reported a significant increase in lung cancer rates; the largest North American study noted an increase that did not reach statistical significance.[3–5] Three recent meta-analyses concluded that patients with SSc have 3–4 fold greater risk of developing lung cancer with males having a higher standardized incident ratio than females.[6–8]

Prior studies of SSc associated lung cancer have identified the presence of the Scl-70 antibody and interstitial lung disease (ILD) as potential risk factors for lung cancer.[9–11] However, radiographic abnormalities and details of pulmonary function testing (PFTs) were not reported. Previous studies describing the histopathology have reported one third to one half of cancers are adenocarcinomas, however, no studies have described the molecular profiles of these cancers, stage at presentation, or outcome.[9,10]

In this cross-sectional analysis, we identify 17 patients with SSc and lung cancer and provide the first description of the immunohistochemical and molecular profile of SSc associated lung cancers.[12,13] In addition, we report the stage of lung cancer at presentation and outcome. Potential risk factors for the development of lung cancer are re-examined, and an analysis of chest imaging and pulmonary function is undertaken.

## Materials and Methods

### Case ascertainment

The Northwestern University Institutional Review Board granted study approval (STU00075739) and waived the need for informed consent because of the retrospective nature of the study and the data were analyzed anonymously. The Northwestern Memorial Healthcare Electronic Data Warehouse (EDW), an electronic repository of health data, was queried for patients >18 years old with ICD-9 diagnostic codes for SSc or SSc related conditions and for lung cancer or pulmonary nodules. The charts identified were reviewed for confirmation of a diagnosis of SSc by a board certified rheumatologist using the American College of Rheumatology criteria for classification of SSc.[14] Patients with mixed connective tissue disease and localized forms of SSc were excluded from analysis. In cases with slides available for review, lung cancers were confirmed by a board certified pathologist (KR) with expertise in pulmonary pathology. In three additional cases, lung cancers were confirmed by review of pathology reports. Those patients meeting both the criteria for SSc and lung cancer were included as cases.

### Data collection

Data abstracted from the medical record included demographics, date of SSc disease onset as defined by first non-Raynaud’s phenomenon diseases manifestation, date of lung cancer diagnosis defined as the date of pathologic diagnosis, chest computed tomography (CT) reports, pulmonary function tests (PFT), serologic markers for SSc, tobacco history (> 5 pack year) and exposure history. PFTs done at Northwestern Memorial Hospital (NMH) were performed in accordance with the American Thoracic Society/European Respiratory Society (ATS/ERS) criteria.[15] All PFT data were reviewed by a board certified pulmonologist (JED) and interpreted according to the ATS/ERS guidelines for interpretation.[16] Chest CT scans were reviewed by a pulmonary radiologist (RA) and classified based on ATS/ERS criteria as consistent with nonspecific interstitial pneumonitis (NSIP), usual interstitial pneumonitis with honeycomb fibrosis (UIP), fibrosis not otherwise specified (nos), or emphysema.[17] Histopathologic and immunohistochemical data were reviewed by a pathologist with an expertise in pulmonary disease in cases with available primary data. Adenocarcinomas were classified according to the International Association for the Study of Lung Cancer (IASLC)/ATS/ERS International Multidisciplinary Lung Adenocarcinoma Classification.[18] The mutational analysis was performed on the deoxyribonucleic acid (DNA) samples extracted from surgically resected, biopsied, or cytologic specimens of lung adenocarcinomas by polymerase chain reaction (PCR) and TrimGens’s Shifted Termination Assay (TrimGens, Sparks, MD) for Kirsten-RAS (KRAS) and Qiagen pyromark 24 sequencer (Valencia, CA) for epidermal growth factor receptor (EGFR) analysis.[19,20]

### Statistical Methods

Descriptive statistics were applied. Means, medians, standard deviations and ranges were reported for numeric date. Categorical data were summarized using frequencies and proportions. Risk factors were assessed by comparison between cases and the Northwestern Scleroderma Patient Registry (579 patients). Cancer characteristics were compared with data from the National Institutes of Health, National Cancer Institute, SEER Cancer Statistics (NCI SEER).[21] Proportions were compared using two tailed Fisher Exact Test. Results were exploratory and no attempt to control for Type I error was made.

## Results

### Demographics

Initial EDW query identified 301 cases meeting criteria for review, and of these 112 cases had lung cancer. Eighty of these cases had Raynaud’s syndrome without SSc, 24 had localized scleroderma, and 8 had other non-SSc connective tissue diseases. Seventeen met the inclusion criteria, 14 females (82%) and 3 males (Table 1). Eight of the 17 cases were in the Northwestern Scleroderma Patient Registry, and 16/17 were seen in the Northwestern Scleroderma Program. The prevalence of lung cancer in the registry was 8/579 (1.4%). The mean age of SSc onset was 48.1 ±12.8 years and the mean age at lung cancer diagnosis was 63.1 ± 8.4 years. In two patients the lung cancer diagnosis predated SSc onset, and in the remaining patients SSc onset preceded lung cancer by 17.3 years (range 2.0 to 43.1 years).

**Table 1:** Demographics, smoking history and SSc subtype and serology.

Case number	Gender	Age at SSc Onset (years)	Age at Lung Cancer (Years from SSc Onset to Cancer)	Smoking History (pack-years)	Years Cessation	SSc Subtype	Serology
1	M	62	74.3 (12.3)	-	-	lcSSc	RNA pol
2	F	61	63 (2.0)	-	-	lcSSc	Scl-70
3	F	39	51.1 (12.1)	-+(10)	21	dcSSc	Scl-70/ RNA pol
4	F	57	66.1 (9.1)	+ (50)	0	dcSSc	Neg
5	F	49	57.1 (8.1)	-	-	lcSSc	Scl-70
6	F	46	59.2 (13.2)	+ (15)	15	lcSSc	ACA
7	F	31	61.9 (30.9)	-	-	lcSSc	Scl-70
8	F	48	76.6 (28.6)	+ (33)	28	lcSSc	Neg
9	F	33	53 (20)	+ (35)	0	SSc/PPM	NA
10	F	63	61.1 (-1.9)	+ (40)	0	lcSSc	ACA
11	F	62	64 (2.0)	+ (80)	2	lcSSc	ACA
12	F	33	76.1 (43.1)	+ (23)	13	lcSSc	NA
13	F	33	51.7 (18.7)	-	-	dcSSc	Scl-70
14	M	41	63 (22)	+ (20)	29	lcSSc	Scl-70
15	M	74	72 (-2)	+ (75)	7	lcSSc	ACA
16	F	36	54 (18)	-	-	lcSSc	ACA negative*
17	F	50	69 (19)	+ (33)	20	lcSSc	Scl-70

NA- not assessed/available

lcSSc- limited cutaneous systemic sclerosis

dcSSc- diffuse cutaneous systemic sclerosis

SSc/PPM- Systemic sclerosis and polymyositis overlap

ACA- anticentromere antibody

SCL-70- anti-SCL 70 antibody

RNA Pol- anti-RNA polymerase III antibody

* SCL-70 not assessed

A history of smoking was identified in 11/17 cases (64.7%) compared with 43% of patients in the registry (p = 0.09). The average exposure was 37.6± 22.8 pack-years (py). Three patients were actively smoking at the time of lung cancer diagnosis; the remainder had stopped between 2 and 29 years prior to diagnosis. Two patients had occupational exposure to chemical solvents. One patient received chemotherapy for lymphoma and one had radiation and chemotherapy for a head and neck cancer. Prior to cancer diagnosis, two patients had been treated with cyclophosphamide, one with a tumor necrosis factor inhibitor, one with azathioprine, and three with mycophenolate mofetil.

### Systemic Sclerosis

Systemic sclerosis was classified as limited cutaneous (lc) in 13 cases (76%), diffuse cutaneous (dc) in 3 (18%) and SSc/polymyositis overlap in 1 (Table 1). This distribution of disease is similar to that in our SSc registry (63%, 36% and 1% for lc, dc and SSc/myositis overlap respectively) (p = 0.14). Results of serologic testing were available for 15 of 17 cases: 13/15 (87%) antinuclear antibody positive, 7/14 (50%) scl-70 antibody (Scl-70) positive, and 4/15 (27%) anticentromere antibody (ACA) positive. RNA Polymerase III antibody titer (RNA pol) was measured in 10 cases and was positive in 2 (20%). The distribution of positive serologic markers in our registry was 24%, 25% and 22% for ACA, Scl-70 and RNA pol respectively, similar to that reported in the literature.[22] The Scl-70 positive cases were significantly more in cases compared with our registry patients (p = 0.04).

### Pulmonary Findings

**Fig 1:**
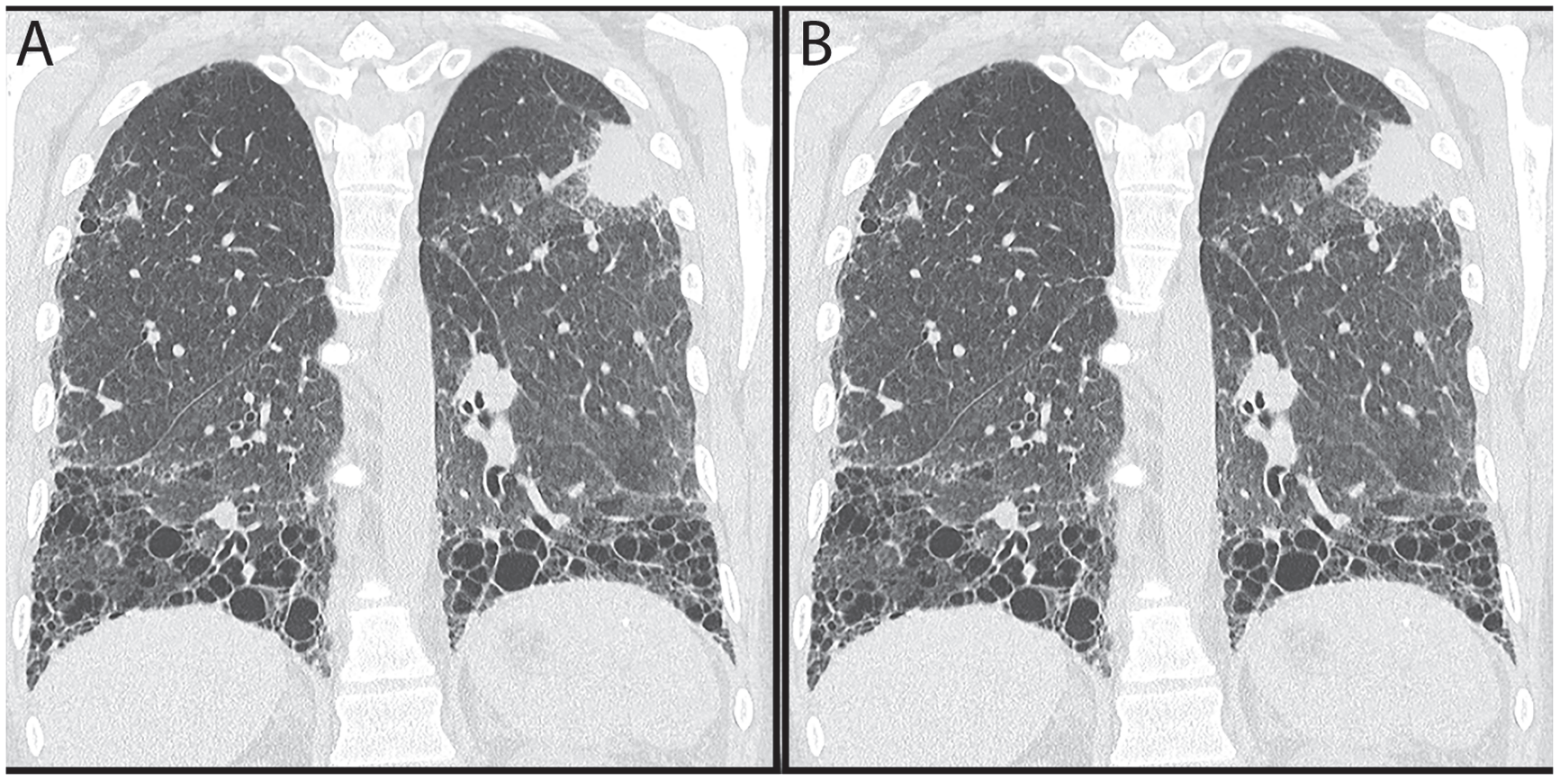
Computed Tomography showing (A) a left upper lobe lung mass with UIP basilar predominant fibrosis and (B) a left lower lobe consolidation (lung cancer) with NSIP.

Sixteen of 17 cases had chest CT imaging available for review, 15 were performed at NMH, 11 were high resolution chest CT (HRCT) (Table 2). The median number of CT scans prior to diagnosis was 3 (range 0–6). Independent review by a pulmonary radiologist identified scleroderma associated lung involvement in 12/15 cases: 9 classified as NSIP, 2 as UIP, 1 as combined fibrosis nos and emphysema (Fig. 1). Thirteen of 16 cases were found to have pulmonary nodules preceding the diagnosis of lung cancer; an additional 2 had cancers diagnosed based on the initial Chest CT scan, and in 1 case available imaging occurred after cancer resection.

**Table 2:** Pulmonary function testing and Computed Tomography findings.

Case number	FVC (% pred)	FEV1 (% pred)	DLco (% pred)	PFT Interpretation	CT pattern
1	84	82	58	Isolated Reduction in DLco	NSIP
2	59	62	41	Restriction	UIP
3	70	73	69	Restriction	NSIP
4	119	106	54	Isolated Reduction in DLco	NA
5	43	44	55	Restriction	NSIP
6	85	87	72	Normal	NSIP
7	80	92	84	Normal	-
8	87	57	56	Obstruction	Fib nos/EMPHY
9	82	83	36	Isolated Reduction in DLco	NSIP
10	62/82*	66	32	Obstruction	EMPHY
11	67	41	41	Obstruction	EMPHY
12	60	65	50	Restriction	NSIP
13	79	90	84	Normal	NSIP
14	61	64	41	Restriction	UIP
15	84	81	46	Isolated Reduction in DLco	NSIP
16	59	64	41	Restriction	NSIP
17	112	116	72	Normal	NSIP

*Total lung capacity, in all other cases FVC and TLC were similar

NSIP- non-specific interstitial pneumonia

UIP- usual interstitial pneumonia

Fib nos- fibrosis not otherwise specified

EMPHY- emphysema

NA- not assessed/available

All cases had PFTs available for review, 14 were performed at NMH. Six had restrictive impairments (mean forced vital capacity 59%+/-8.7 predicted, range 43–70%) with low diffusing capacity for carbon monoxide (Dlco). All of these cases had radiographic NSIP or UIP. Three cases had obstructive impairments with low DLco (mean forced expiratory volume at 1 second (FEV1) 55%+/-12.7 predicted, range 41–66%), 2 of whom had radiographic emphysema and 1 who had combined emphysema and fibrosis. Four cases had isolated reductions in DLco, 3 of these cases had NSIP and 1 had no radiographic images available. Four cases had normal PFTs, 1 had no radiographic abnormalities but 3 had NSIP. The DLco was reduced in 13 of 16 cases (44% predicted +/- 10.4). Of the six cases with radiographic NSIP who did not have restrictive physiology on PFTs at the time of cancer diagnosis, 2 subsequently developed restriction.

### Cancers

**Fig 2:**
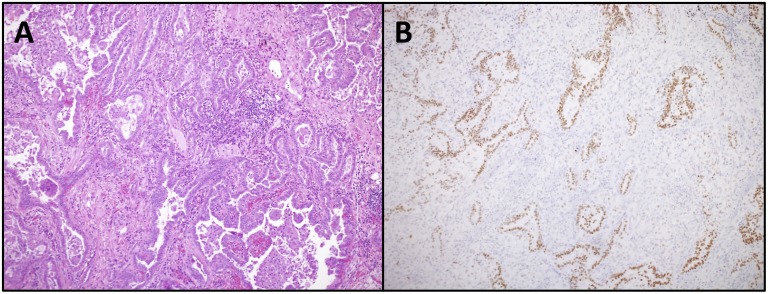
Sections from the tumor of one of the patients with hematoxylin and eosin staining shows infiltrating tumor glands with acinar, lepidic and focal papillary features with surrounding areas of dense fibrosis and scattered chronic inflammation (A). These tumor cells stain positive with TTF-1 (B).

Two of the cancers were discovered as incidental masses on imaging, 5 were incidental nodules followed over time, and 10 cases had imaging after complaint of worsening dyspnea, change in cough, or new weight loss. Thirteen of the cancers were adenocarcinoma, 1 adenosquamous, 1 squamous, 1 small cell, and 1 large cell neuroendocrine (Table 3). Of the patients with adenocarcinoma, 4 had a predominant lepidic pattern, 5 had an acinar predominant pattern, and 1 patient had adenocarcinoma with micropapillary features. TTF-1 immunostain was performed in 7 of the adenocarcinomas and stained tumor cells diffusely in 5, focally in 1, and was negative in 1 (Fig. 2). In the 7 resected lung cancers, 5 had visceral pleural invasion (4 stage Ib and 1 with IIIb). Tumor sizes ranged from 1.5 cm to 7.5 cm. Ten of 14 adenocarcinomas underwent molecular mutational profiling: 2/8 had KRAS mutation, 1/10 had EGFR mutation, and 0/5 had ALK rearrangement.

**Table 3:** Tumor characteristics and prognosis.

Case number	Histopathology (Type)	Stage at diagnosis	Tumor Size (cm)	Solitary or Multiple Nodules	TTF-1 staining	Molecular features	Alive/Deceased (Years post-diagnosis)
1	Adenocarcinoma	Ib	5.5	Solitary	Focal positivity	KRAS(G12T), EGFR (WT)	Alive (1.1 years)
2	Adenocarcinoma	IV	2.0	Multiple	Positive	EGFR (WT), ALK (no rearrangement)	Deceased (0.2 years)
3	Adenosquamous	Ia	2.5	Solitary	Positive	KRAS (WT), EGFR (WT), ALK (no rearrangement)	Alive (1.5 years)
4	Small Cell	Limited	1.5	Solitary	NA	NA	Alive (0.9 years)
5	Adenocarcinoma	IV	2.6	Multiple	Positive	EGFR (WT), ALK (no rearrangement)	Deceased (1.3 years)
6	Adenocarcinoma	IV	1.5	Solitary	NA	KRAS (WT), EGFR (WT), ALK (no rearrangement)	Deceased (0.7 years)
7	Adenocarcinoma	Ia	2.1	Solitary	NA	NA	Alive (7.0 years)
8	Adenocarcinoma	IV	2.5	Multiple	Positive	KRAS (WT), EGFR (WT), ALK (no rearrangement)	Deceased (2.0 years)
9	Adenocarcinoma	Ib	3.2	Solitary	Positive	KRAS (WT), EGFR (WT)	Deceased (3.4 years)
10	Adenocarcinoma	Ib	3.4	Solitary	Positive	KRAS(WT), EGFR (E746 mutation in exon 19)	Deceased (3.7 years)
11	Adenocarcinoma	IV	2.8	Multiple	Positive	NA	Deceased (0.2 years)
12	Adenocarcinoma	Ia	1.6	Solitary	NA	NA	Deceased (1.2 years)
13	Adenocarcinoma	IIIa	5.4	Multiple	NA	KRAS(WT), EGFR (WT)	Deceased (0.1 years)
14	Large Cell Neuroendocrine	IIIb	4.4	Solitary	Negative	NA	Deceased (0.3 years)
15	Adenocarcinoma	IIb	7.5	NA	NA	NA	Alive (1.6 years)
16	Adenocarcinoma	Ib	5.3	Solitary	Negative	KRAS (mutation in exon 12/13)	Alive (<0.5 years)
17	Squamous	Ib	6.4	Solitary	Negative	NA	Alive (<0.5 years)

NA- not assessed/available

WT: wild type

Eight of the non-small cell lung cancers presented at stage I (50%), 3 at Ia and 5 at Ib disease. One case was stage IIb, 2 cases were stage III, and 5 cases were stage IV. NCI SEER data for cancers of the lung and bronchus diagnosed between 2004 and 2010 (n = 268,097) reported that 15% presented with localized disease (stage I or II), 22% with regional disease and 57% with distant metastases. Thus, significantly more of our cases (56%) presented with early stage, localized, disease (p = 0.01). Nine of the 16 cases were alive at the time of data collection, with all seven deaths attributed to progression of cancer or cancer therapy related death. The 5 years survival among the stage I cases that had completed follow-up for 5 years was 25% versus expected survival of 54%.

## Discussion

The prevalence of lung cancer in the Northwestern Scleroderma Registry (1.4%) is at the lower range of previously published studies of SSc cohorts, but it is higher than the estimated 2011 prevalence in the US population (SEER). Lung cancer was diagnosed at a younger age than is reported in the SEER database (63yo vs 70yo). The high female predominance of cases likely reflects the high proportion of female patients enrolled in the Northwestern Scleroderma Registry (84% female). Only 65% of our cases had a smoking history, compared to an 80% smoking rate reported in females with lung cancer,[23] and not significantly higher than the smoking rate in our registry. This further supports SSc itself, independent of tobacco, as a risk factor for lung cancer.

Recent publications have shown associations between SSc serology and cancer risk, with the possibility of an immunologic mechanism linking autoantibodies to RNA poI and tumorigenesis.[24,25] The role of RNA pol has not been assessed in prior lung cancer studies. While the low rate of RNA pol measurement in our cohort limits analysis, we did not find a higher rate of this marker in our cases compared with patients in our registry or than reported in the literature. The Scl-70 antibody has also been explored as a risk factor for the development of lung cancer with variable results.[9,10] We did find a significantly higher proportion of positive Scl-70 in our cases than in our registry.

Interstitial lung disease has been reported as a risk factor for lung cancer development in some but not all SSc case series.[9–11] In our cases, a high proportion of patients had ILD, though not all demonstrated restrictive physiology on pulmonary function testing at the time of cancer diagnosis. Thus the high rate of radiographic ILD in our cases may reflect our practice of CT screening for ILD, resulting in recognition of either early ILD prior to the development of restrictive physiology on PFTs or very mild ILD that would otherwise not prompt diagnostic imaging. The variable results reported in the literature may thus reflect different practice patterns with respect to Chest CT screening. This may also be relevant to the stage of lung cancer at presentation in our cohort. Our cases were diagnosed at an earlier stage of disease compared with the general population of lung cancer as represented by the NCI SEER database. The percent of patients diagnosed in stage I (50%) is comparable to patients who underwent low dose Chest CT screening as part of the National Lung Screening Trial (NLST) wherein 58% were stage 1 at diagnosis.[26] Lung nodules were present on 81% of our patients’ CT scans prior to the diagnosis of cancer, and an additional 12.5% were diagnosed with lung cancer based on their initial CT scan. This highlights the importance of screening Chest CT scans in patients with SSc who are actively smoking or who have a history of smoking, and is an area that needs further investigation. Importantly, the NLST included only patients with a tobacco exposure of at least 30 py, an exposure only achieved in 7 of the 11 smokers in this study. While similar to the NSLT screened patients, lung cancers in our cases were found at an earlier stage, unfortunately, this did not confer a survival advantage as it did in the NLST study. In fact, SSc associated lung cancer seems to portend a worse prognosis as indicated by the 5 year survival rates in the early stage lung cancers in our cohort.

The histological pattern of the SSc associated lung cancer in the reported literature is mixed, with an equal predominance of adenocarcinoma and squamous cell carcinoma, and with few cases of small cell carcinoma.[7] In contrast, the majority of patients in our cohort had adenocarcinoma with a predominance of lepidic and acinar patterns. Chronic diffuse fibrotic lung diseases can often show areas of epithelial proliferations including honeycomb change and peribronchiolar metaplasia with and without atypia. These changes have been postulated as an underlying mechanism for the development of adenocarcinoma.[27] Indeed, differentiating these atypical epithelial proliferations from well-differentiated adenocarcinoma can be challenging and features such as monomorphic population with cytologic atypia, architecture complexity of the glandular proliferations, and an invasive growth pattern can be helpful in making an unequivocal diagnosis of adenocarcinoma in such cases. It may be speculated that the predominance of adenocarcinoma in our cases was due to the prevalence of underlying SSc associated inflammation and fibrosis as many of our patients had radiographic evidence of either NSIP or fibrosis on Chest CT scans. In another diffuse lung disease, idiopathic pulmonary fibrosis, an increased risk of lung cancer has been found[28] yet the predominant histopathology has not been adenocarcinoma.[29] Only one study showed a predominance of adenocarcinoma in a subset of cases whose cancers were located within a region of fibrotic lung.[30]

This is the first report of molecular markers in SSc associated lung cancers. The rate of KRAS mutation (2/8) is consistent with the rate of KRAS mutation found in adenocarcinomas in our institution.[31] Only 1/10 had EGFR mutation, which is also in keeping with the expected rate.[32] However interpretation of this data is limited by the small number of cases with molecular markers performed.

## Conclusion

Patients with SSc are at increased risk for the development of lung cancer. While the lung cancers in our cases were diagnosed at an early stage, likely due to the practice of screening for ILD, this did not confer a survival advantage; indeed mortality was higher than anticipated in those with stage I cancers. While our results need to be further explored, they do suggest that screening CT scans should be undertaken for all smokers or former smokers regardless of pack year history and that aggressive follow-up of pulmonary nodules in patients with SSc, regardless of tobacco history, is warranted. The high proportion of adenocarcinoma seen in our study is surprising and different from that reported in the literature. Molecular profiling appears to be similar to lung cancers in general.
