# Correction: Early Stage Lung Cancer Detection in Systemic Sclerosis Does Not Portend Survival Benefit: A Cross Sectional Study

**DOI:** 10.1371/journal.pone.0124225

**Published:** 2015-04-02

**Authors:** The PLOS ONE Staff

In Fig. 1, panel B is incorrect. Please see the corrected Fig. 1 here.

**Fig 1:**
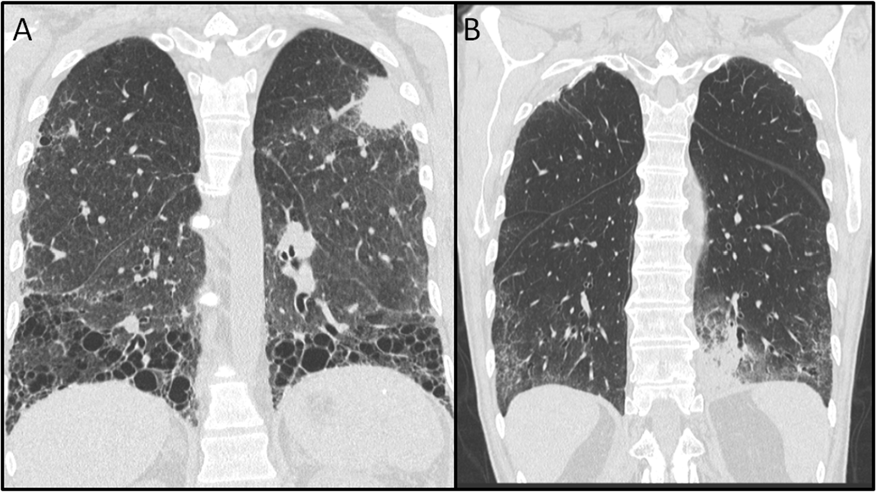
Computed Tomography showing (A) a left upper lobe lung mass with UIP basilar predominant fibrosis and (B) a left lower lobe consolidation (lung cancer) with NSIP.

## References

[1] Katzen JB, Raparia K, Agrawal R, Patel JD, Rademaker A, Varga J, et al (2015) Early Stage Lung Cancer Detection in Systemic Sclerosis Does Not Portend Survival Benefit: A Cross Sectional Study. PLoS ONE 10(2): e0117829 doi: 10.1371/journal.pone.0117829 25689302 10.1371/journal.pone.0117829 PMC4331488

